# Structural Basis of the Immunological Cross-Reactivity between Kiwi and Birch Pollen

**DOI:** 10.3390/foods12213939

**Published:** 2023-10-27

**Authors:** Ricarda Zeindl, Annika L. Franzmann, Monica L. Fernández-Quintero, Clarissa A. Seidler, Valentin J. Hoerschinger, Klaus R. Liedl, Martin Tollinger

**Affiliations:** 1 Institute of Organic Chemistry, Center for Molecular Biosciences Innsbruck (CMBI), University of Innsbruck, 6020 Innsbruck, Austria; ricarda.zeindl@uibk.ac.at (R.Z.); annika.franzmann@student.uibk.ac.at (A.L.F.); 2 Institute of General, Inorganic and Theoretical Chemistry, Center for Molecular Biosciences Innsbruck (CMBI), University of Innsbruck, 6020 Innsbruck, Austria; monica.fernandez-quintero@uibk.ac.at (M.L.F.-Q.); clarissa.seidler@uibk.ac.at (C.A.S.); klaus.liedl@uibk.ac.at (K.R.L.)

**Keywords:** PR-10, nuclear magnetic resonance, protein structure, *Actinidia deliciosa*, *Actinidia chinensis*

## Abstract

Allergies related to kiwi consumption have become a growing health concern, with their prevalence on the rise. Many of these allergic reactions are attributed to cross-reactivity, particularly with the major allergen found in birch pollen. This cross-reactivity is associated with proteins belonging to the pathogenesis-related class 10 (PR-10) protein family. In our study, we determined the three-dimensional structures of the two PR-10 proteins in gold and green kiwi fruits, Act c 8 and Act d 8, using nuclear magnetic resonance (NMR) spectroscopy. The structures of both kiwi proteins closely resemble the major birch pollen allergen, Bet v 1, providing a molecular explanation for the observed immunological cross-reactivity between kiwi and birch pollen. Compared to Act d 11, however, a kiwi allergen that shares the same architecture as PR-10 proteins, structural differences are apparent. Moreover, despite both Act c 8 and Act d 8 containing multiple cysteine residues, no disulfide bridges are present within their structures. Instead, all the cysteines are accessible on the protein’s surface and exposed to the surrounding solvent, where they are available for reactions with components of the natural food matrix. This structural characteristic sets Act c 8 and Act d 8 apart from other kiwi proteins with a high cysteine content. Furthermore, we demonstrate that pyrogallol, the most abundant phenolic compound found in kiwi, binds into the internal cavities of these two proteins, albeit with low affinity. Our research offers a foundation for further studies aimed at understanding allergic reactions associated with this fruit and exploring how interactions with the natural food matrix might be employed to enhance food safety.

## 1. Introduction

Kiwi fruit has gained global popularity due to its remarkable health benefits [1]. Kiwis exhibit an extraordinarily high content of vitamin C, an essential dietary component for humans. Ensuring an adequate intake of vitamin C is crucial for reducing the risk of chronic diseases including cardiovascular diseases and certain types of cancer. Indeed, kiwi fruit is especially recognized for its heart-healthy properties, acting as a vasodilator that enhances blood circulation and flow [2]. However, the growing popularity of kiwi fruit has also raised health and food safety concerns. For a subset of individuals, the consumption of kiwi can lead to allergic reactions with diverse clinical manifestations and the potential for severe reactions [3]. Allergic symptoms to kiwi fruit often manifest as oral allergy syndrome (OAS), marked by tingling or itching sensations in the mouth, lips, and throat. Moreover, kiwi allergic individuals may also experience cutaneous symptoms such as hives and angioedema, and in severe cases, anaphylaxis can occur [4]. Most allergic reactions to kiwi typically occur within minutes of consuming either the fruit itself or a food product containing kiwi. The wide range of these reactions and their severity underscores the critical need for precise diagnosis and effective management of kiwi allergy. However, while advances in allergen identification and diagnosis have significantly improved our understanding of kiwi allergy, the primary strategy for preventing allergic reactions remains avoidance of the fruit.

Numerous proteins found in kiwi fruit have been registered by the World Health Organization and the International Union of Immunological Societies (WHO/IUIS) Allergen Nomenclature Sub-committee [5]. These proteins encompass thirteen allergens from green kiwi fruit (*Actinidia deliciosa*), designated as Act d 1 through Act d 13 [6,7], and three allergens from gold kiwi fruit (*Actinidia chinensis*), namely Act c 5 [8], Act c 8 [6], and Act c 10 [9]. Allergic reactions to kiwi fruit are notably common among individuals with pollen allergies [10]. Approximately 48% of individuals sensitized to the allergen Bet v 1, which is present in birch pollen, experience allergic symptoms upon consuming kiwi fruit [11]. This immunological cross-reactivity arises from the initial allergic sensitization to Bet v 1, followed by the subsequent recognition of structurally similar proteins in kiwi fruit by Bet v 1-specific IgE antibodies. Bet v 1 is a pathogenesis-related class 10-like (PR-10-like) protein. It shares the same three-dimensional fold as PR-10 proteins, which are commonly found in various fruits and vegetables [12]. As a result, more than 70% of individuals allergic to birch pollen eventually develop allergies to one or more of these food sources at some point in their lives [11].

Two PR-10 proteins in kiwi fruit have been identified as food allergens: Act c 8 from gold kiwi and Act d 8 from green kiwi, with each protein having a molecular weight of 17 kDa. Both proteins have been reported to exhibit IgE binding in the sera of individuals allergic to birch pollen, and their cross-reactivity with Bet v 1 has been studied in detail [13]. The precise quantity of these allergens in kiwi fruit remains uncertain, but tissue prints of both gold and green kiwi fruits suggest that these allergens are more abundant in the fruit’s skin than in the pulp.

The amino acid compositions of Act c 8 and Act d 8 differ from other PR-10 proteins in terms of the relative abundance of cysteines. Act c 8 contains seven cysteines, accounting for 4.4% of all amino acids, while Act d 8 possesses four cysteines, which corresponds to 2.6% of all amino acids. These values are atypical for PR-10 proteins, for which a low content of sulfur-containing amino acids has been noted [14]. Cysteines can significantly impact protein structures by forming intramolecular disulfide bridges that stabilize the three-dimensional fold. The structural and immunological characteristics of disulfide bridges in various kiwi protein allergens have been investigated in detail [15,16,17]. Cysteines that are exposed on the protein’s surface may also form intermolecular disulfide bridges or participate in reactions with components of the natural food matrix, including secondary metabolites such as polyphenols [18,19,20].

The three-dimensional structures of the two kiwi allergens Act c 8 and Act d 8 have not been reported so far. In a previous study, we described the recombinant expression of these two proteins and derived their secondary structure composition [21]. We now report the three-dimensional structures of Act c 8 and Act d 8, determined in solution by nuclear magnetic resonance (NMR) spectroscopy. We present a binding study of Act c 8 and Act d 8 with pyrogallol, a phenolic compound that is highly abundant in kiwi fruit [22], and characterize the oxidation states of cysteines in these proteins in detail. Knowledge of the three-dimensional structure of these allergens and their ligand binding characteristics serve as the basis for understanding the immunological cross-reactivity between kiwi and birch pollen at the molecular level [23,24].

## 2. Materials and Methods

Recombinant Protein Expression and Purification. The plasmid generation for Act c 8 (isoform Act c 8.0101, GenBank nucleotide code AM489567.1, protein code CAM31908.1) and Act d 8 (isoform Act d 8.0101, GenBank nucleotide code AM489568, protein code CAM31909) have been described previously [21]. For recombinant expression of the allergens, transformation was carried out using the *E. coli* strain BL21(DE3) Star (Invitrogen). A starter culture (20 mL) of Luria Bertani medium, supplemented with 25 µg/mL kanamycin, was inoculated with a single bacterial colony. After incubation for 8 h at 37 °C and 200 rpm, 20 µL of the starter culture was transferred into 100 mL of M9 minimal medium and incubated overnight under the same conditions. Subsequently, an aliquot of the overnight culture was precipitated by centrifugation at 2000× *g* and resuspended in 1 L of M9 minimal medium, enriched with ^15^NH_4_Cl (1 g/L) or ^13^C_6_-d-glucose (3 g/L) and supplemented with 25 µg/mL kanamycin, to give a cell density of 0.1 (at 600 nm). The culture was incubated at 37 °C and 200 rpm until the cell density reached 0.5–0.6, at which point protein expression was induced by adding isopropyl-β-d-1-thiogalactopyranoside (1 mM). After 3 h (37 °C), the cells were harvested by centrifugation at 4 °C and 4600× *g* (40 min), resuspended in buffer containing 0.5 M urea, 25 mM imidazole, and 0.1% Triton X-100, and stored at −80 °C until use.

For lysis, the cells were thawed and treated with lysozyme (10 µg/mL) and DNAse (1 µg/mL) on ice for 40 min and passed through a French press. Following a 45 min centrifugation at 16,000× *g* and 4 °C and filtration using a 0.45 µm syringe filter, the lysate was loaded in an anion exchange column (Resource Q 6 mL, GE Healthcare, Chicago, IL, USA). Protein was eluted with a NaCl gradient over 30 mL from 0 to 50% in 25 mM TrisHCl buffer (pH 7.5) at a flow rate of 2 mL/min. Fractions containing protein were concentrated to 1.5 mL (Amicon Ultra 3 kDa molecular weight cutoff, Merck Millipore), loaded onto a size exclusion column (HiLoad 16/600 Superdex 75 pg, GE Healthcare), and isocratically eluted at 1 mL/min using 20 mM sodium phosphate buffer (pH 6.9). SDS-PAGE gel electrophoresis was used to monitor all purification steps. No reducing agents were added before or during purification.

Structure Determination. The resonance assignments for both proteins with all corresponding NMR experiments have been previously reported and have been deposited under the accession numbers 50811 and 50812 for Act d 8 and Act c 8, respectively, at the Biological Magnetic Resonance Data Bank [21]. Additional NMR experiments for structure determination, ^1^H-^15^N-NOESY-HSQC and ^1^H-^13^C-NOESY-HSQC, were recorded at 25 °C on a 500 MHz Agilent DirectDrive 2 spectrometer equipped with a room temperature probe. NMR samples contained 0.5 mM ^15^N- or ^13^C/^15^N-labeled protein in a 20 mM sodium phosphate buffer, pH 6.9, supplemented with 10% D_2_O. NMR buffers were degassed, but no reducing agent was added. All experimental data that were used for resonance assignments and structure determination of Act d 8 and Act c 8 were recorded in this buffer [25]. All NMR data were processed with NMRPipe [26] and visualized and interpretated using the CcpNMR software package [27].

Assigned NOE cross-peaks were classified into four subclasses according to their peak intensities and used as distance restraints. These subclasses were defined by their upper restraint distance boundaries: 6 Å (weak), 5 Å (light), 4 Å (medium), and 2.8 Å (strong). Dihedral angle restraints (Φ and Ψ) were obtained from the program TALOS+ [28]. Using the program XPLOR-NIH, version 2.52 [29], a set of 500 structures were determined in 3000 steps at an initial temperature of 7000 K, followed by 10,000 cooling steps. 

The 20 structures with the lowest energy were further refined in explicit solvent. Using the tleap tool of the AmberTools20 package [30], the structures were soaked in cubic water boxes of TIP3P water molecules with a minimum wall distance of 10 Å to the protein [31]. The structures were described with the AMBER force field 14SB [32]. After solvent relaxation [33], simulated annealing calculations with a Langevin thermostat (collision frequency: 2 ps^−1^) [34] and a Berenden barostat (relaxation time: 2 ps) [35] were performed. Hydrogen bonds were constrained with the SHAKE algorithm [36], and a Van der Waals cutoff of 10 Å was used along with the particle-mesh Ewald method for long-range electrostatics [37]. A simulated annealing scheme of 50 ns with a time step of 1 fs for each structure was performed using the NOE distance restraints. The protein structure validation software (PSVS) suite was used to validate the refined structures [38]. Internal cavity volumes and surface hydrophobicity were determined using PyMOL [39]. 

MMTS Modification. Labeling of ^15^N Act c 8 with methyl methanethiosulfonate (MMTS) was performed according to a modified protocol by Religa [9]. A total of 180 nmol of ^15^N-labeled Act d 8 in 20 mM sodium phosphate buffer pH 6.9 was incubated with a sevenfold molar excess of MMTS overnight at 37 °C. The reaction was quenched by washing the protein three times with fresh 20 mM sodium phosphate buffer pH 6.9. The MMTS-modified ^15^N-labeled Act c 8 sample was concentrated to 0.2 mM in 450 μL and used to record a two-dimensional ^1^H-^15^N-HSQC spectrum. The sample was subsequently used for analysis by mass spectroscopy. For this purpose, the sample was washed five times with 100 mM ammonium acetate pH 6.8, followed by five times with water. The protein solution was diluted to an appropriate amount for analysis by mass spectrometry.

NMR Diffusion Experiments. A stimulated-echo pulsed-field-gradient NMR experiment was used to examine the translational diffusion of Act c 8 (concentration: 0.2 mM) in the identical buffer employed for structure determination, at 25 °C, both when DTT (dithiothreitol) was absent and when it was present (concentration: 2 mM). Experimental details were identical to those reported for Bet v 1 [40].

Pyrogallol Binding. We used 0.2 mM ^15^N-labeled samples of Act c 8 and Act d 8 in 20 mM sodium phosphate buffer pH 6.9 for ^1^H-^15^N-HSQC titration experiments with pyrogallol. These experiments were recorded on a 700 MHz Bruker Avance Neo spectrometer equipped with a Prodigy CryoProbe at 25 °C. Pyrogallol (1 M in 20 mM sodium phosphate buffer pH 6.9, supplemented with 10% D_2_O, was added stepwise to the protein samples. The molar ratios were 1:2, 1:8, 1:16, 1:32, 1:64, and 1:128 protein/pyrogallol. For each protein, a series of identical ^1^H-^15^N-HSQC spectra was recorded. Residue-specific chemical shift perturbations (CSPs) were used to determine dissociation constants, *K*_d_, as described [41].

## 3. Results

### Three-Dimensional Structures of Act c 8 and Act d 8

**Figure 1:**
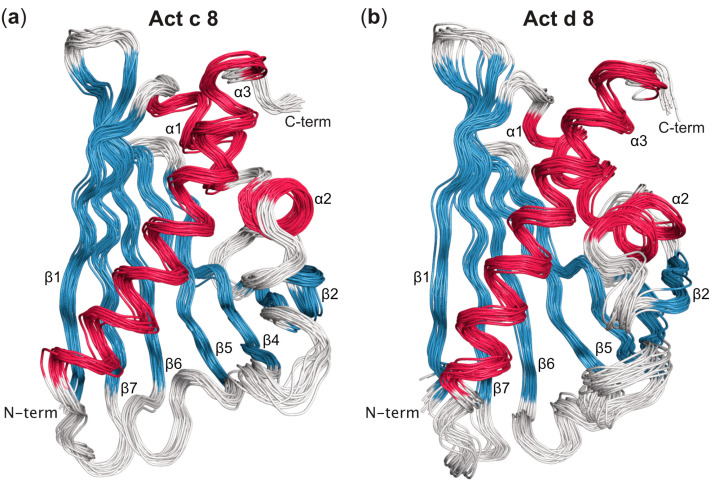
Overlay of the 20 lowest energy structures of (**a**) Act c 8 from gold kiwi fruit (isoform Act c 8.0101, PDB accession code 8QHI) and (**b**) Act d 8 from green kiwi fruit (isoform Act d 8.0101, PDB accession code 8QHH). Secondary structure elements are labeled and colored in red (α-helices) and blue (β-strands). N- and C-termini are indicated.

The three-dimensional structures of the kiwi allergens Act c 8 and Act d 8 consist of a curved, seven-stranded antiparallel β-sheet (designated as β1–β7) and three α-helices, adopting the characteristic PR-10 fold (Figure 1). Specifically, a pair of short helices, α1 and α2, are positioned above the β-sheet, acting as V-shaped support for helix α3 at the C-terminus. Together with the curved β-sheet, these helices shape a large internal cavity. The NMR-based structural ensembles of both Act c 8 and Act d 8 exhibit well-defined secondary structure elements and a high degree of conformational homogeneity. The heavy-atom root-mean-square deviation (RMSD) values for the 20 lowest energy structures are 0.5 Å for Act c 8 and 0.6 Å for Act d 8 (Table S1). As observed in other PR-10 proteins, solvent-exposed loops connecting secondary structural elements and the protein’s C-terminus exhibit increased conformational variability [42,43]. 

The backbone RMSD between the Act c 8 and Bet v 1 (isoform Bet v 1.0101), computed as 1.3 Å, reveals the high similarity in the three-dimensional structures of these two allergens. When comparing Act d 8 to Bet v 1, a somewhat higher RMSD of 2.4 Å is computed. Structural differences between Act d 8 and Bet v 1 are evident for the loops connecting secondary structure elements. Notably, the loop between strands β5 and β6 in Act d 8 is one residue shorter than in Bet v 1, resulting in a different spatial orientation. In addition, the C-terminal tail following helix α3 in Act d 8 is both shorter by two residues and structurally distinct from the C-terminal tail in Bet v 1. Consequently, excluding loops and termini, the RMSD values between the secondary structure elements β1–β7 and α1–α7 in the kiwi PR-10 allergens Act c 8 and Act d 8 with Bet v 1 reduce to 1.2 Å and 1.6 Å, respectively.

**Figure 2:**
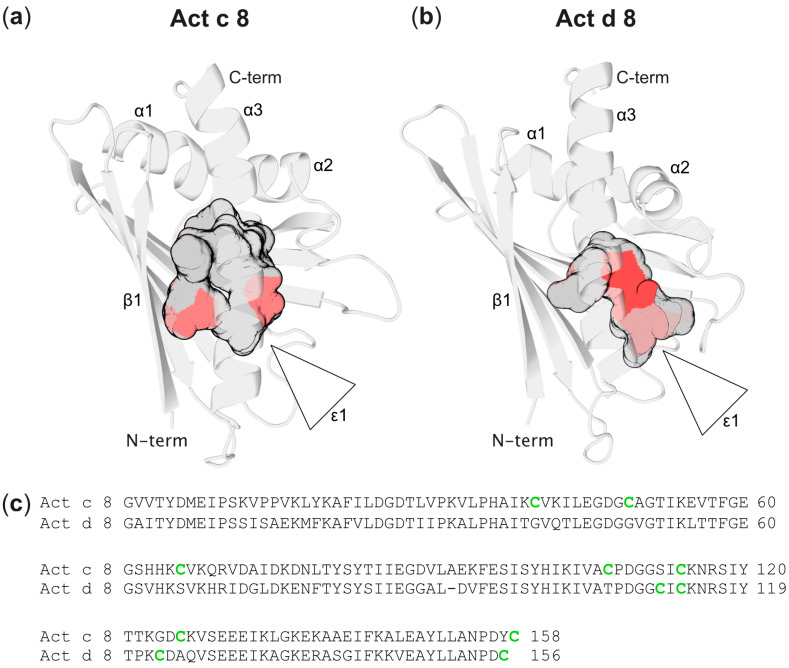
Backbone and internal cavity of (**a**) Act c 8 and (**b**) Act d 8. Entrances to the cavities (ε1) are indicated. The internal cavities are color-coded, with grey indicating hydrophobic surfaces and red representing hydrophilic surfaces. (**c**) Sequence alignment of Act c 8 and Act d 8. Cysteines are shown in green.

As in other PR-10 proteins, the internal cavities in Act c 8 and Act d 8 exhibit inner surfaces that are formed by both hydrophobic and hydrophilic amino acids (Figure 2). Notably, the hydroxyl-bearing side chains of numerous tyrosines in the central β-sheet (Tyr5, Tyr81, Tyr83, Tyr100, and Tyr120) convey a polar character to the surface of the cavity of Act c 8 and Act d 8. Together with phenylalanines Phe22, Phe58, and Phe144, these aromatic amino acids constitute a substantial feature of the inner cavity in both kiwi allergens, a characteristic that appears to be conserved in other PR-10 proteins as well [43]. In both Act c 8 and Act d 8, ligands can access the cavity through an amphiphilic entrance (ε1), which is delimited by the N-terminal end of helix α3 and the connecting loops between strands β3 and β4, strands β5 and β6, and strand β7 with helix α3. 

Act c 8 contains a total of seven cysteines, representing 4.4% of all amino acids, while Act d 8 features four cysteines, accounting for 2.6% of the total amino acids in each protein (Figure 2). In the three-dimensional structure of Act c 8, except for Cys114, all cysteines (Cys40, Cys49, Cys66, Cys107, Cys126, and Cys158) are located on the protein’s exterior, with their side chains exposed and accessible to the solvent. These cysteines are distributed across the protein surface, with pair-wise minimum distances exceeding 12 Å in all instances, well outside the range required for disulfide bridge formation. Cys114, on the other hand, is located in the protein’s interior, where its side chain contributes to the internal cavity. In Act d 8, Cys123 and Cys156 are situated on the protein’s outer surface, on opposite sides of the protein, while Cys111 and Cys113 are present in strand β7, but their side chains are positioned too far apart to allow for the formation of a disulfide bond.

This is consistent with the NMR chemical shifts of β-carbons in cysteine side chains, which serve as diagnostic indicators of disulfide bridge formation and allow for the differentiation between cysteines in their reduced (free) and oxidized (disulfide-bonded) states [44]. For Act c 8 and Act d 8, the cysteine side chain β-^13^C chemical shifts are within the range of 26.0–32.3 ppm [21], which is characteristic of the reduced state of the side chain thiol (β-^13^C chemical shifts of 40.7 ± 3.8 ppm are expected in disulfide bridges). This indicates that all cysteines in these two PR-10 proteins do not participate in intramolecular or intermolecular disulfide bridge formation. 

To obtain information about the solvent-accessibility of the cysteine residues in the kiwi allergen, Act c 8 was treated with methyl methanethiosulfonate (MMTS), a reagent that reacts with exposed cysteine side chains to form S methylthiocysteine. In electospray ionization (ESI) mass spectra (Figure S1), we detected a mass shift, Δm, of 320.9569 Da, suggesting that all seven cysteine residues in this particular protein underwent chemical modification (Δm = 46.087 Da is the expected value for attaching a methylthio group to a single cysteine thiol group). This observation is further supported by ^1^H-^15^N-HSQC spectra of Act c 8, which revealed that the resonances of the seven cysteine residues were affected by treatment with MMTS (Figure S1). These data suggest that all cysteines in this PR-10 protein are accessible to the solvent and available for chemical modification by MMTS. 

In order to determine the oligomerization state of Act c 8 under the experimental conditions employed in our study (20 mM sodium phosphate buffer, pH 6.9), we conducted pulsed-field-gradient NMR-diffusion experiments. We obtained a value of 18.4 ± 0.6 Å for the hydrodynamic radius of Act c 8, a value comparable to the hydrodynamic radius of monomeric Bet v 1 (20.1 Å) under similar experimental conditions [40]. This is consistent with our observation that, using the same buffer, Act c 8 elutes from a size exclusion column with a retention time that is virtually identical to that of Bet v 1. Notably, the addition of 2 mM dithiothreitol (DTT) as a reducing agent had no measurable effect on the hydrodynamic radius of Act c 8. Taken together, these data demonstrate that the kiwi allergen Act c 8 exists as a monomer in solution and does not engage in disulfide-bridge mediated dimerization under the conditions used for structure determination.

**Figure 3:**
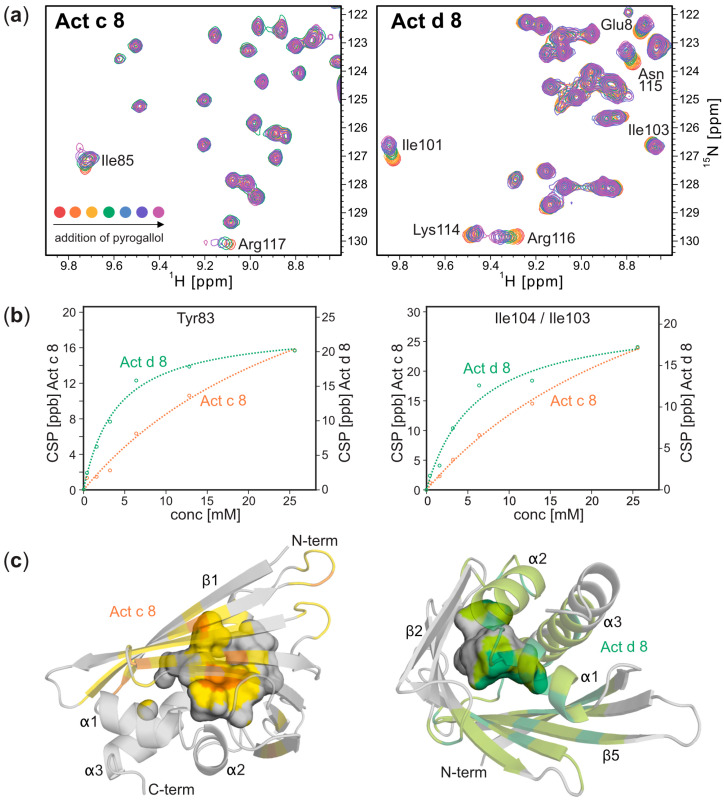
Pyrogallol binding to Act c 8 and Act d 8. (**a**) Sections from backbone amide ^1^H–^15^N HSQC spectra of Act c 8 (left) and Act d 8 (right), 0.2 mM in the presence of variable amounts (up to 128-fold excess) of pyrogallol. (**b**) Pyrogallol binding curves (ligand-induced ^1^H–^15^N chemical-shift perturbations, CSP) for two representative amino acid residues in Act c 8 (orange) and Act d 8 (green). *K*_d_ values were derived from these data by nonlinear least-squares fitting, indicated by dashed lines. (**c**) Amino acids that interact with pyrogallol in Act c 8 (left) and Act d 8 (right) are marked in orange and green on their respective structures. The shades of light orange and light green represent a 4 Å expansion around these amino acids. The inner surface of the cavity is shown and colored accordingly.

Next, we probed the binding of pyrogallol, which is the most abundant phenolic compound in kiwi fruit [22], to Act c 8 and Act d 8 by NMR spectroscopy. For both proteins, the addition of pyrogallol leads to a gradual change in the chemical shifts of the backbone amide ^1^H-^15^N-resonances, in accordance with an intermediate-to-fast binding process on the NMR chemical-shift timescale, as is typically observed for low-affinity binding (Figure 3). Amino acids in the β-sheet and in all three α-helices were affected by pyrogallol clustering around the internal cavity of the PR-10 fold, suggesting that this phenolic compound binds to the protein’s interior. Pyrogallol had a more pronounced effect on the ^1^H-^15^N resonances in Act d 8 than in Act c 8. Quantitative analysis of the NMR data yielded dissociation constants, *K*_d_, of 5 mM for Act d 8 and 30 mM for Act c 8 (median values), indicating that both allergens bind pyrogallol weakly.

## 4. Discussion

**Figure 4:**
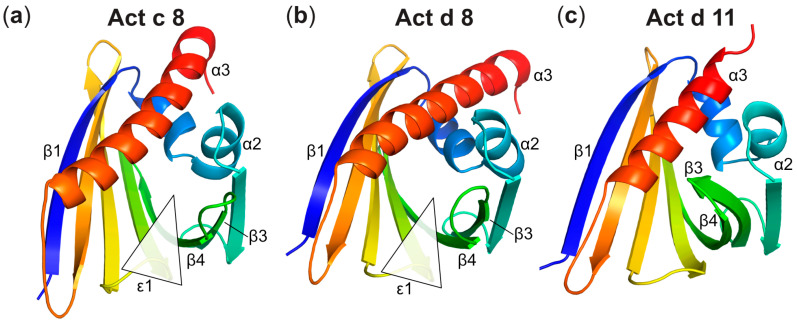
Comparison of the PR-10 proteins (**a**) Act c 8 (PDB accession code: 8QHI), (**b**) Act d 8 (isoform Act d 8.0101, PDB accession code: 8QHH), and (**c**) Act d 11 (PDB accession code: 4IHR), a member of the family of ripening-related proteins (RRP). Secondary structure elements are colored from blue (N-terminus) to red (C-terminus). For Act c 8 and Act d 8, the entrances to the cavities (ε1) are indicated. Act d 11 represents the ligand-bound state of this protein, as an unidentified ligand is present within the cavity and an opening to the cavity is not evident.

The three-dimensional structures of the two PR-10 allergens from kiwi, Act c 8 from gold kiwi and Act d 8 from green kiwi, are highly similar to each other and also to the structure of the sensitizing allergen from birch pollen, Bet v 1. This is in accordance with the observed immunologic cross-reactivity between these proteins. For proteins from the Bet v 1 family, IgE antibody binding typically involves surface regions formed by amino acids that come together in the protein’s folded, three-dimensional structure (conformational epitopes). Consequently, structural similarity is a prerequisite for immunologic cross-reactivity. Regarding the two kiwi PR-10 allergens Act d 8 and Act c 8, structural differences concern the orientation of the two short helices α1 and α2, and the long C-terminal helix α3, as evident in Figure 4. Similar observations have previously been reported for other PR-10 proteins [14]. In addition, the loop between strands β5 and β6 in Act d 8 is one residue shorter than in Act c 8, resulting in a slightly more constrained local geometry. Nevertheless, despite these differences, similar immunologic characteristics have been reported for these two allergens [13]. 

It is instructive to compare the structures of Act c 8 and Act d 8 with the kiwi allergen Act d 11, a member of the ripening-related protein (RRP) family. Proteins from this family display a similar overall fold as PR-10 proteins but low sequence similarity [45]. Nevertheless, Act d 11 displays IgE co-recognition with allergens from the PR-10 family and Bet v 1, implying a potential overlap in IgE epitopes between these allergens [46]. Act d 11 shares sequence identities with the two kiwi PR-10 proteins Act c 8 and Act d 8 of 25% and 24%, respectively, while Act c 8 and Act d 8 are 70% identical to each other. Regarding their three-dimensional structures, various differences between Act d 11 and the two PR-10 proteins are evident, the most prominent being the length of the C-terminal helix α3 (Figure 4). This helix comprises between 24 and 25 amino acids in Act c 8 and Act d 8 (as well as in Bet v 1), but it consists of only 17 amino acids in Act d 11. Another striking feature of Act d 11, as noted previously [45], pertains to the conformation of the segment encompassing strands β3 and β4, along with the connecting loop. In Act d 11, this segment packs against helix α3, whereas in Act c 8 and Act d 8, this part of the structure is positioned farther away from the helix, opening up entrance 1 to the internal cavity. Interestingly, in PR-10 and PR-10-like proteins, this particular segment consistently exhibits conformational flexibility in a solution when no ligand is bound inside the cavity [47]. The crystal structure of Act d 11 represents the ligand-bound state of this protein, as an unidentified ligand is present within the cavity, and the entrance to the cavity is closed. An opening to the internal cavity of Act d 11 is not evident in the available crystal structure. 

Allergens in green kiwi fruit (Act d 1 through Act d 13) and gold kiwi fruit (Act c 5, Act c 8, and Act c 10) contain on average 4.0% cysteines, significantly exceeding the occurrence in most organisms [48]. To date, structural data for eight kiwi proteins that are also allergens are available: Act d 1 [49], Act d 2, Act c 5 and Act d 5 [50,51], Act d 6 [52], Act c 10 [53], Act d 11 [45], and the two PR-10 proteins Act c 8 and Act d 8 from this study. 

Except Act d 11, which contains a single cysteine residue, all of these kiwi allergens qualify as cysteine-rich proteins. The thaumatin-like protein Act d 2 contains 16 cysteines, constituting 4.4% of all amino acids, with all cysteines participating in intramolecular disulfide bridges. This structural feature is conserved in thaumatin-like proteins, conveying structural stability to their three-dimensional scaffold [54]. It was shown that disulfide bonds in Act d 2 play a crucial role in the resistance of this allergen to degradation by proteases that are typically found in the gastrointestinal tract [16]. The two proteins Act c 5 and Act d 5, characterized by a distinctive three-dimensional structure unique to kiwillins, contain 14 cysteine residues (7.4% of all amino acids), all of which form stabilizing disulfide bridges. Likewise, Act c 10, a member of the non-specific lipid transfer protein (nsLTP) family, has all 8 cysteines (7.0% of all amino acids) participating in disulfide bridges, a characteristic structural feature of nsLTPs. In the cysteine protease actinidin Act d 1, all cysteines form disulfide bridges except for the active site cysteine, and in the pectin methylesterase inhibitor Act d 6, all but one cysteine participates in disulfide bridges, stabilizing the three-dimensional fold of this kiwi allergen [15]. Neither Act c 8 nor Act d 8 display disulfide bridges in their three-dimensional structures. 

PR-10 proteins are widely distributed in the plant kingdom, where they are upregulated in response to various kinds of abiotic and biotic stress. The biological role of PR-10 proteins entails the binding and, possibly, the transport of ligand molecules within their internal cavity [14]. Indeed, PR-10 proteins promiscuously bind a vast variety of amphiphilic, low-molecular-weight ligands with different affinities, such as fatty acids, flavonoids, phenolic acids, and cytokinins. Our experimental data show that the two kiwi PR-10 proteins Act c 8 and Act d 8, too, are capable of binding amphiphilic ligands to their internal cavities. Whether and how ligand binding to PR-10 allergens is related to their immunologic properties is currently under debate [24].

We previously showed that the two kiwi PR-10 proteins Act d 8 and Act c 8 exhibit conformational flexibility across their entire structure in the absence of ligands, which is reminiscent of “conformational breathing” [47]. Indeed, the ability to bind and release ligand molecules appears to be intricately connected to the PR-10 scaffold’s conformational flexibility, as it enables ligands to access the internal cavity [55,56]. For Bet v 1, experimental observations have shown that binding a low-molecular-weight ligand reduces conformational flexibility, leading to the rigidification of the PR-10 protein scaffold and resulting in a more compact three-dimensional structure [40]. The data presented in this work form the basis for further investigations into the effects of interactions with ligand molecules on the biophysical properties of PR-10 proteins.

## 5. Conclusions

We showed that two kiwi allergens, Act c 8 and Act d 8, exhibit the canonical PR-10 fold and possess the ability to bind ligands within their internal cavities. Act c 8 and Act d 8 are characterized by a high abundance of cysteines, contrasting the low content of sulfur-containing amino acids that has been noted for other PR-10 proteins. Interestingly, all reactive cysteines are accessible on the outer (or inner) surface of Act c 8 and Act d 8. This characteristic feature may well be of relevance for interactions of these proteins with the surrounding natural food matrix. Chemical modifications involving the cysteine thiols can result in (partial) coverage of the allergen’s surface, potentially decreasing the accessibility of conformational epitopes for antibody binding, and rendering these allergens less immunoreactive. The high abundance of reactive cysteines in Act c 8 and Act d 8 thus holds potential for the development of strategies to increase the food safety of kiwi fruit by promoting chemical modifications with the natural food matrix during food treatment and processing.

## Data Availability

The data used to support the findings of this study can be made available by the corresponding author upon request.

## Supplementary Materials

The following Supporting Information can be downloaded at: https://www.mdpi.com/article/10.3390/foods12213939/s1, Figure S1: ESI-MS and NMR spectra of Act c 8 before and after addition of MMTS; Table S1: Summary of restraints used for structure determination of Act c 8 and Act d 8 and structure refinement statistics.
