# Supplementary material for: Structural Basis of the Immunological Cross-Reactivity between Kiwi and Birch Pollen
Source: Foods. 2023 Oct 27;12(21):3939. doi: 10.3390/foods12213939 (PMC10649968; doi:10.3390/foods12213939)
Supplement: Supplementary file 1 [file foods-12-03939-s001.zip › foods-2672902-supplementary.pdf]

SUPPLEMENTARY MATERIAL TO:

Structure and Ligand Binding of the Kiwi Allergens Act c 8 and Act d 8

Ricarda Zeindl [1], Annika L. Franzmann [1], Monica L. Fernández-Quintero [2], Clarissa A. Seidler [2], Valentin J. Hoerschinger [2], Klaus R. Liedl [2] and Martin Tollinger [1]

[1] Institute of Organic Chemistry, Center for Molecular Biosciences Innsbruck (CMBI), University of Innsbruck, Innsbruck, Austria
[2] Institute of General, Inorganic and Theoretical Chemistry, Center for Molecular Biosciences Innsbruck (CMBI), University of Innsbruck, Innsbruck, Austria

Table S1. Summary of restraints used for NMR structure determination of Act c 8 and Act d 8 and refinement statistics.

	Act c 8	Act d 8
**PDB ID**	**8QHI**	**8QHH**
**Experimental Restraints**		
NOE-based distance restraints	4241	1602
intraresidue [i = j]	1285	327
sequential [|i - j|= 1]	817	547
medium range [1 < |i - j| < 5]	753	335
long range [|i - j| ≥ 5]	1386	393
Dihedral angle restraints	276	256
Hydrogen bond restraints	178	136
Total number of restraints	4695	1994
Total restraints per residue	29.7	12.8
Total long range restraints per residue	8.8	2.5
**Restraint Violations**		
average distance violation	1.74 Å	1 07 Å
maximal distance violation	2.02 Å	1.22 Å
average dihedral angle violation	28.56°	12.11°
maximal dihedral angle violation	32.44°	13.40°
**RMSD Values** [1]		
backbone atoms	0.4 Å	0.5 Å
heavy atoms	0.5 Å	0.6 Å
bond lengths	0.013 Å	0.013 Å
bond angles	2.2°	2.0°
**Ramachandran Plot Statistics** [1]		
most favored regions	88.6%	93.1 %
allowed regions	11.0%	5.6 %
disallowed regions	0.4%	1.2 %

[1] Determined using the Protein Structure Validation Software package PSVS.

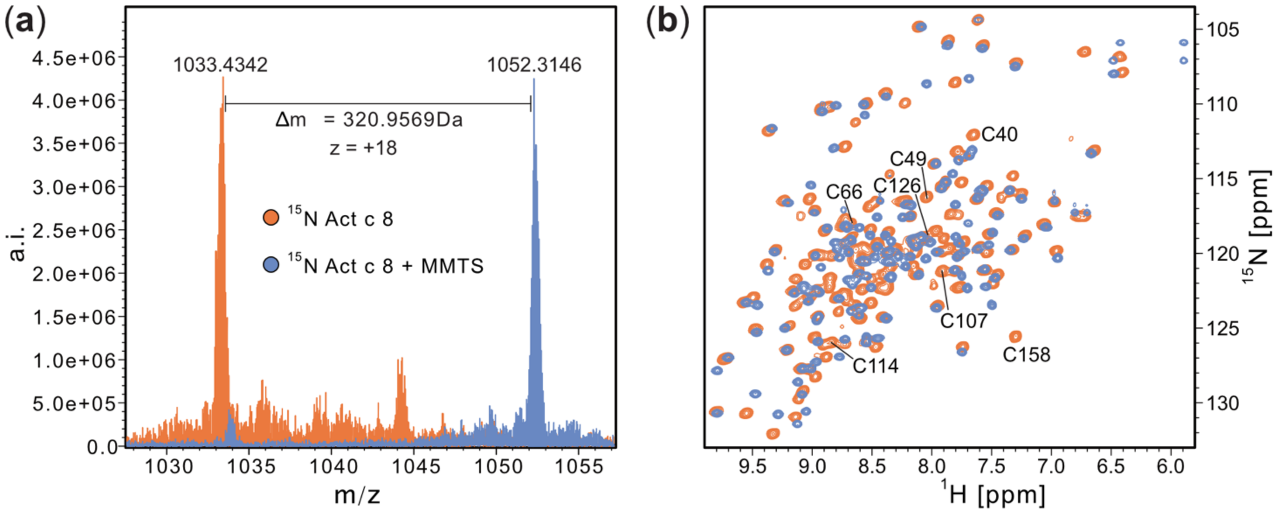

Figure S1: (**a**) ESI-MS spectra of ^{15}N-labeled Act c 8 before (orange) and after (blue) addition of methyl methanethiosulfonate MMTS, charge state z = +18. The observed mass shift, Δm, of 320.9569 Da corresponds to the covalent attachment of methylthio groups to all seven cysteines in this protein. (**b**) 700 MHz ^{1}H-^{15}N-HSQC of ^{15}N-labeled Act c 8 before (orange) and after (blue) addition of MMTS. The backbone amide resonances of the seven cysteine residues in Act c 8 are labeled in the spectrum that was obtained before MMTS was added. The chemical shifts of all cysteines are affected by MMTS treatment, along with numerous other resonances. For the MMTS modified protein resonances were not assigned. Experimental conditions: 0.4 mM ^{15}N-labeled Act c 8 in 20 mM $Na_xH_{(3-x)}PO_4$, pH 6.9, 9 % D_2O, 25 °C.
